# Frozen elephant trunk reconstruction for right-sided aortic arch with aberrant left subclavian artery and aneurysm of the descending aorta: a case report

**DOI:** 10.1186/s13019-016-0479-y

**Published:** 2016-05-05

**Authors:** John Kokotsakis, Omar A. Jarral, Leanne Harling, Panteleimon Tsipas, Thanos Athanasiou

**Affiliations:** Evaggelismos Hospital, Athens, Greece; Department of Surgery and Cancer, Imperial College London, London, W2 1NY UK

**Keywords:** Aortic arch, Endovascular procedures

## Abstract

**Background:**

A 59-year old man being investigated for back pain was found to have aneurysmal dilatation of a right-sided aortic arch and descending thoracic aorta together with an aberrant left subclavian artery.

**Case Presentation:**

He underwent repair of this utilising the frozen elephant trunk technique, which dealt with all three pathologies in one-stage. He made an unremarkable recovery and was discharged home on the 8th post-operative day.

**Conclusions:**

This case report further demonstrates the flexibility and safety of the frozen elephant trunk in dealing with complex aortic pathology as a single-stage procedure.

## Background

A right-sided aortic arch is an uncommon congenital lesion occurring in 0.05–0.10 % of the population. It is often associated with an aberrant left subclavian artery, and patients are particularly prone to developing aneurysm and dissection [1]. The optimal approach to this condition requires meticulous planning and depends on the extent of aneurysm formation. We report the successful single stage repair of this vascular anomaly using the frozen elephant trunk technique.

## Case presentation

**Fig. 1:**
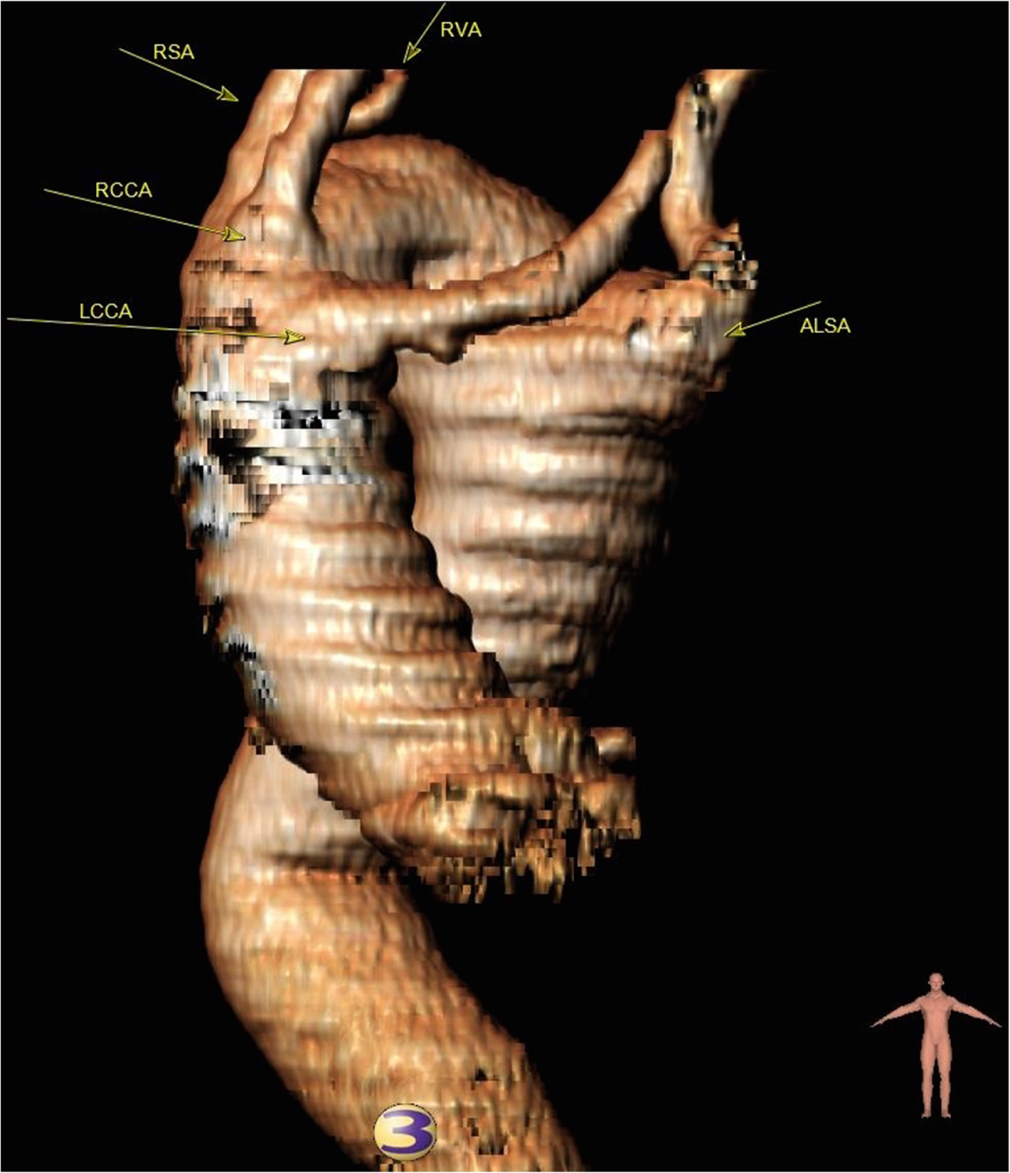
Computer generated images of aorta

A 59-year old male was referred to our clinic with a six-month history of back pain. His medical history included hypertension and a 60-pack year history of smoking. Physical examination including assessment of peripheral pulses was unremarkable. Chest radiograph revealed a laterally displaced trachea and a right-sided aortic arch. Computerized tomographic angiography (CTA) confirmed a right-sided aortic arch (4.1 cm) and an aneurysmal descending thoracic aorta (DTA, 6.8 cm at its largest point) located in the right hemi-thorax, which returned to normal size below the diaphragm. The root and ascending aorta were not aneurysmal. An aberrant left subclavian artery (ALSA) arose from a Kommerell’s diverticulum (3.5 cm) at the junction between the distal arch and the proximal DTA. The head and neck vessels arose individually from the arch in the following order: left common carotid artery (LCCA), right common carotid artery (RCCA), right subclavian artery (RSA) and finally the ALSA as mentioned above (Fig. 1). Coronary angiography, echocardiography and carotid doppler scan were unremarkable. After multi-disciplinary team discussion it was decided to proceed with the frozen elephant trunk technique.

After induction of anaesthesia, arterial lines were placed in the left femoral and both radial arteries. A lumbar cerebrospinal fluid drain was placed in order to manipulate cord perfusion pressure. A soft guidewire was placed in the right femoral artery (RFA) and advanced in to the arch. A median sternotomy was performed with left supraclavicular extension in order to expose the distal LSA. The right axillary artery (RAA) was exposed with an infraclavicular incision. After heparinization, a 8 mm graft was anastomosed to the RAA through this incision on the underside of the artery. The other end of the graft was tunnelled through the right pleural space in to the pericardial cavity and the arterial line (bifurcated) of the cardiopulmonary bypass (CPB) circuit was connected to this end. Examination of the aorta revealed no evidence of dissection or rupture.

After going on to CPB a right superior pulmonary vein vent was placed and the patient was cooled to 25 °C. During cooling the head and neck vessels were dissected out, and upon fibrillation the ascending aorta was clamped and transected just above the sinotubular junction. The heart was arrested using direct cold crystalloid cardioplegia. Subsequent shots were delivered retrogradely every 20 min. Once the target temperature was reached, CPB was arrested and the ascending aorta and part of the arch (up to 2 cm before the origin of the RSA) were resected. The LCCA and RCCA were transected 1 cm distal to their origin and the RSA was ligated at its origin from the aortic arch. Perfusion catheters were inserted in to the LCCA, the RCCA, and together with the RAA cannula already in place, continuous bilateral selective antegrade cerebral perfusion (SACP) was started.

At this point the soft guidewire in the RFA was exchanged for a stiff one. A hybrid stent-graft (E-vita OPEN PLUS 40 × 160 mm, Jotec Inc., Hechingen, Germany) was advanced in to the DTA in an antegrade fashion. The proximal landmark for stent deployment was just distal to the origin of the RSA. The incorporated polyester graft was cut back and sutured to the transected distal arch with interrupted horizontal mattress sutures (3-0 Prolene, Ethicon, Somerville, NJ) reinforced with a strip of Teflon. A sterilized bronchoscope enabled the distal part of the stent graft to be checked under direct vision.

**Fig. 2:**
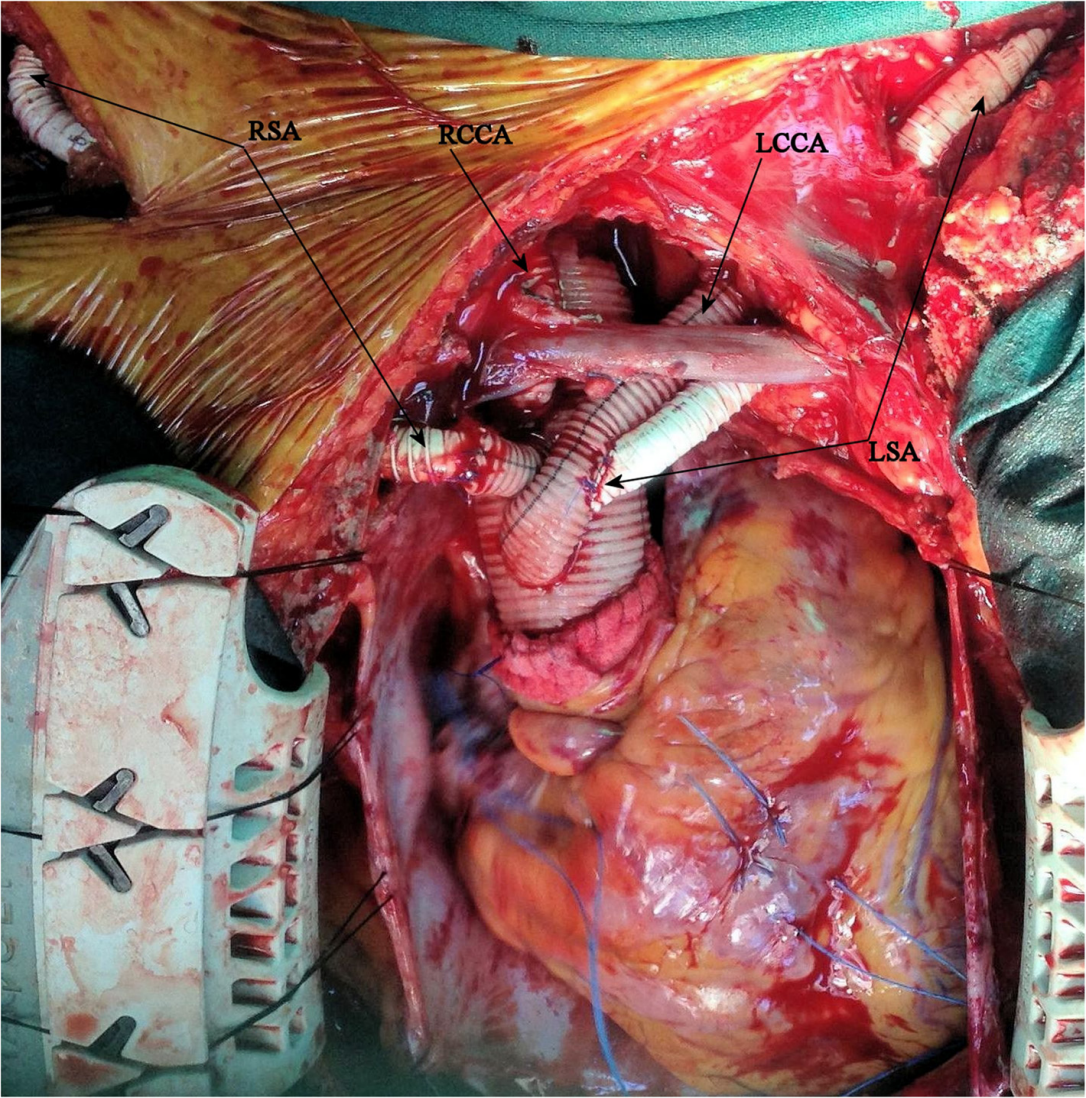
Intraoperative photo

Attention was then turned to the ascending aorta where a vascular prosthesis with four side branches (30 mm Tetrabranch, Jotec Inc.) was anastomosed distally to the arch cuff (comprising the native aorta and the E-vita prosthesis/Teflon strip as described above) using a continuous suture (3-0 Prolene, Ethicon). Systemic perfusion was re-established by connecting the arterial line of the CPB circuit to the 4^th^ side branch of the vascular prosthesis. The 3^rd^ side branch (8 mm) was passed under the innominate vein and was anastomosed end-to-end to the RCCA (after removal of the perfusion catheter) with a continuous suture (5-0 Prolene, Ethicon). The proximal anastomosis of the main graft to the sinotubular junction was then performed using a continuous suture (3-0 Prolene, Ethicon) with an external strip of Teflon. Rewarming was commenced; a de-airing drill completed and the cross-clamp was removed. The 1^st^ side branch (10 mm) was passed under the innominate vein and anastomosed end-to-end to the LCCA (after removal of the perfusion catheter) with a continuous suture (5-0 Prolene, Ethicon). Arterial inflow to the RAA graft, which had been tunnelled to sit in the pericardial cavity, was then stopped and this end of the graft was anastomosed end-to-end to the 2^nd^ side branch(8 mm) of the prosthesis using a continuous suture (5-0 Prolene, Ethicon). A separate 8 mm spiral graft was then anastomosed end-to-side to the ALSA in the left supraclavicular area and connected proximally end-to-side to the 1^st^ branch of the main vascular prosthesis (Fig. 2). The ALSA was ligated proximal to the origin of the left vertebral artery.

After complete rewarming the patient was separated from CPB uneventfully. The bypass times were as follows: CPB (160 mins), SACP (90 mins) and, distal ischaemic time (50mins). Following haemostasis, the synthetic grafts were covered with a strip of pericardium to exclude them from the sternal wound prior to routine closure. The patient was extubated 6 h following the operation and discharged on the 8^th^ post-operative day at which point he was mobile and neurologically intact. Twelve-month follow-up with CTA confirmed satisfactory appearance of the reconstruction with absence of endoleak.

## Discussion

Right-sided aortic arch with ALSA is a rare congenital abnormality frequently associated with aneurysm and dissection. Whilst appropriate surgical indications are not yet clearly established, intervention in the asymptomatic is usually recommended for aneurysms over 3 cm [1]. Meticulous pre-operative imaging and planning are essential. Current treatment options are tailored to the anatomical features (e.g., type I or II right-sided arch and the degree of aneurysm formation) of each case and include: right (or left) thoracotomy with replacement of the descending thoracic aorta and/or reconstruction of the ALSA, sternotomy and debranching of the arch vessels with subsequent thoracic endovascular aneurysm repair (TEVAR), sternotomy and total arch replacement with insertion of elephant trunk and subsequent TEVAR, total arch replacement with subsequent thoracotomy for DTA replacement, arch replacement and DTA replacement through the clamshell approach, and left carotid to left subclavian artery bypass with ligation or coiling at the origin of the ALSA [1].

In this case we utilised the frozen elephant trunk technique in one-stage to deal with three pathologies: arch and descending aortic aneurysm together with an ALSA originating in a Kommerell’s diverticulum. To our knowledge, this is only the third such report in the literature [2, 3]. We consider it to be a safe and effective technique given the excellent outcome this patient achieved, especially as some of the above techniques are associated with very significant morbidity. There are now a number of other devices on the market (e.g., Thoraflex Hybrid), which would allow a similar or more advanced outcome to be achieved. Whilst it could be suggested that TEVAR(after debranching) is a less invasive option, this was not considered due to the acute angle between the distal arch and the DTA, which may have led to difficulty in securing a good proximal landing. Proceeding with TEVAR in such a situation may have led to incomplete treatment of the arch aneurysm and perhaps unwanted complications such as endoleak. A further disadvantage of this approach is that of embolic related complications in patients with extensive atheroma of the distal aorta. In retrospect, we could have simplified our operation further by establishing CPB via arch cannulation and reanastomosing the RSA to the new arch graft directly via the median sternotomy.

## Conclusion

This case report demonstrates the flexibility and safety of the frozen elephant trunk in dealing with right-sided aortic arch aneurysms as a single-stage procedure.

### Consent

Written informed consent was obtained from the patient for publication of this case report and any accompanying images.
